# Digital encounters: Human interactions in mHealth behavior change interventions

**DOI:** 10.1177/20552076211029776

**Published:** 2021-06-30

**Authors:** Ulrika Müssener

**Affiliations:** Department of Health, Medicine and Caring Sciences, Linköping University, Linköping, Sweden

**Keywords:** mHealth, behavior change, interactions, encounters, therapeutic alliance

## Abstract

Digitalization and high mobile phone ownership globally have radically changed communication in all areas of society, including health care. Previous research has shown the effectiveness of behavior change interventions delivered by mobile phones and has highlighted advantages, such as that they require fewer resources than traditional face-to-face interventions and can be delivered at any time. One of the foremost questions pertaining to unsupported digital interventions is whether they can ever be comparable to in-person interventions. Little is known about the therapeutic alliance and the specific qualities of encounters in digital interactions for behavior change. Human interactions in digital interventions and their relationship with outcomes require further investigation. This paper aims to encourage critical reflection and further consideration of mHealth behavior change interventions in a digital age, when even the professional is excluded from the intervention. Questions are raised on the feelings associated with digital therapeutic relationships and how such interactions might affect user’s capacity for behavioral change. Some technological features and human-like considerations for enhancing digital encounters in mHealth interventions are given. Finally, suggestions for future research to facilitate the digital encounter in mHealth behavior change interventions is presented.

## Introduction

### The power of interactions

Interaction between human beings can shape unforgettable experiences that impact emotions, decisions, and even lives. Humans have interacted – human to human and face to face – for thousands of years. Remote communication between individuals has occurred since ancient times, through such means as smoke signals, carrier pigeons and mail couriers, and more recently through telephone and the internet, with a rapid growth of mobile phones in the 1990s. Mobile phones have changed interactions between people as they allow people to interact fast verbally as well as in written form via text messages, images and video. Digitalization and high mobile phone ownership globally have radically changed communication across society, including health care. The portability and capabilities of mobile phones have resulted in a huge increase in the use of mHealth interventions, which is defined by WHO as a medical or public health practice that is supported by mobile devices.^1^ The use of mHealth technologies, as an enabler to self-management and to assist, inform, guide and treat patients with various problems, diseases and disorders, is an intuitive choice for people seeking healthcare services, given the frequent use of mobile phones by approximately 7 billion people, which is 95% of the world’s population.^2^

### mHealth to promote a healthier lifestyle

The prevention and treatment of diseases related to modifiable behavior has been emphasized as a key component of adolescent health worldwide.^3^ Since unhealthy behaviors typically emerge during adolescence, track into adulthood, and commonly co-occur^4–6^ efforts for outreach to young adults are crucial.^7^ Moreover, young people are early adopters of technology and frequent users of mobile phones.^8^ Systematic reviews have investigated the feasibility, acceptability, efficacy^7–13^ of digital interventions for behavior changes, and provides exciting possibilities for health behavior promotion among young adults. Major advantages with mHealth interventions are that they require fewer resources than traditional face-to-face interventions, low cost, can be accessed by much of the population, and can overcome barriers in time, mobility, and geography.^14^ The quality and success of mHealth interventions partially depend on how they have been developed. Guidelines for the development of digital and mobile health interventions now exist,^15^ highlighting the need to examine development processes.^16^ We have previously described the development processes of mHealth interventions targeting health behaviors,^17,18^ and evaluated the effects using randomized controlled trials.^19,20^

It is now time to widen the perspective a little. In the absence of human support, an important question to raise is: can mHealth behavioral change interventions ever be comparable to in-person interventions? The portability and capabilities of mobile phones have profoundly affected interpersonal behavior including the interaction between the client and the professional. Little is known about the specific qualities of digital encounters (digital interactions between a sender behind the intervention and a user). It may be argued that that a failure to address the digital encounter may limit the true potential of mHealth interventions in promoting a healthier lifestyle.

The aim of this paper is to encourage critical reflection and further consideration of the interactions in mHealth interventions targeting behavior change. This paper refers to interventions without human support, accessed via mobile phones. Initially a brief outline of encounters and therapeutic alliance during in-person meetings are given, followed by a reflection of human interactions through digital devices. Thereafter, design considerations, both technological and human-like, that may enhance the digital encounters are highlighted. Lastly, proposals for future research with the potential to facilitate interactions in mHealth interventions are presented.

## The nature of face-to-face interactions

Previous research on face-to-face meetings in the medical field has, during recent decades, concluded that people are affected in different ways by how and with whom they experience encounters. Qualities of a good experience in meetings, such as being listened to, treated with respect and in a reassuring manner might influence clients’ experiences that the medical condition has improved, and in that way lead to more effective treatment or outcomes in the rehabilitation process.^21,22^ Care decisions have, traditionally, been made in the context of a relationship between patient and professional honed through professional training to help empower patients. The original conceptualization of the therapeutic alliance by Bordin^23^ included the following components: a bond between the client and the professional, joint agreement of the tasks directed toward improvement, and agreement on goals. Rogers^24^ defines active components of the therapeutic alliance to be empathic understanding, acceptance, and congruence. There is no easily applicable consensus definition of the therapeutic alliance, but elements include acceptance, mutual trust, alliance, respect, empathy, and genuine relationship between the client and the professional.^25^ The therapeutic alliance in face-to-face therapy has in several meta-analysis and review revealed a positive relationship with treatment outcome.^25,26^ Yet, the role of the therapeutic alliance in the behavioral change process and outcomes among adults in general, and young people in particular, remains unclear.^27^ The encounters between the client and the professional might evoke emotions such as pride and shame; those feelings are very closely linked to self-perception and self-esteem in the individual. Encounters might influence how individuals are able to use their internal resources in a wider sense; for example, clients’ ability to make changes to their lifestyles.^28^

## The human interaction in mHealth interventions

Communication through electronic devices can be synchronous and/or asynchronous, in the later the parties do not receive an immediate response from another or may lack any response at all. Interactions in digital solutions make it harder to read another’s tone of voice and body language, simply because mHealth interventions lack many of the physical and non-verbal cues made available in face-to-face communication, including facial expressions and body language cues as well as tone and prosody of speech. Although therapeutic alliance is used as a key factor in explaining the effects of face-to-face interventions^25^ this concept has received little empirical attention in the mHealth field and is an relatively underrecognized consideration in digital interventions especially in interventions without any human support.^29^ The therapeutic alliance is different when arranged via electronic devices in that there is typically considerably less, or no, contact. According to Tremain et al^29^ simply replacing therapist with program or app in existing measures may fail to account for the complexity of the therapeutic alliance in digital interventions. Results from their narrative review^29^ conclude that a therapeutic alliance can be fostered in mHealth interventions, but that it may have unique, yet-to-be-confirmed characteristics in a digital context.

### Strengths of digital interactions

There is a risk that communication through electronic devices may fail in interpreting human-like qualities, such as empathy, respect, or prudence and that the information provided tends to be too generic, leading to the perceptions of robotic features that hinder the development of a relational bond.^30,31^ mHealth interventions serve a different function from relationships with professionals. Barriers regarding stigma and lack of trust in available treatments, and the potential for mHealth to reduce fears about exposing and embarrassing oneself compared with talking to a person, suggest that the nonhuman element in mHealth interventions could prove helpful^10^ and improve encounters.^32^ Although human support seems beneficial for the therapeutic alliance and the outcome of the treatment, in many cases, human support is not practical, cost effective or even desirable.^29^ A systematic review of Ames et al.^33^ explored clients’ perceptions of communication via the healthcare system through their mobile phones. The results described a range of participants’ preferences for digital health interventions compared with in‐person visits to professionals. People’s opinions differ, and some studies described that participants felt that these interventions provided them with feelings of support and connectedness, as they reported that someone was giving their time (to send them messages), which made them feel that someone was thinking of them and cared about them. Some participants experienced mHealth interventions as more convenient, reliable, flexible, less judgmental, and faster, and stated that they provided more frequent support. Others perceived interacting with a professional in-person as preferable, warmer, and as something with which they were accustomed.^33^ Digital interventions need to be as engaging and trustworthy as possible to effect behavior change. Therefore, in unsupported digital interventions, it becomes important to incorporate other features that resemble a therapeutic relationship between the user and the intervention.

### Components to facilitate interactions in mHealth interventions

There are several design considerations, both technological and human-like, that may enhance the digital encounters. Some examples of technological features and human-like qualities claimed to be best positioned to foster the digital encounters are highlighted next.

In mHealth interventions the ‘professional’ takes up less, or no, space in treatment, i.e., the professional is less present in physical space, but might be more present in terms of availability. mHealth interventions might, if the user requires it, offer more frequent ‘contact’ compared with face to face. Being able to select the frequency of the messages is stressed as one feature that might affect the experience of the intervention as more personalized. There seem to be a fine balance between feeling bombarded and of not receiving enough information or support. Aspects of importance include requesting the time and day for the message(s) to be sent.^33^ Rapid progress in mobile health technologies has enabled the design of just-in-time adaptive interventions,^34^ which may open the possibility to give behavioral support that directly correspond to a need in real-time. However, within and across studies and client groups, there are no consensus as to the ideal timing, or frequency, as this is linked to personal preferences, contextual factors, and the behavior or information the mHealth intervention is trying to target.^33^ Other aspects concern the opportunity to tailor the intervention to meet personal needs, such as reminders and privacy settings, being able to select a preferred language, and being able to select content.^33^ Tailoring needs to provide individuals with support or information that is relevant and fits with his or her situation and needs.^35^ Tailoring, and personalizing content may help foster a digital therapeutic alliance between the user’s personal needs and goals and interactions with the intervention.^36^ Individuals seem more motivated to engage with and process information more thoroughly if the content is personally relevant and meaningful.^37^ Using the individual’s name^36^ is another way to personalize mHealth interventions, however, opinions differ regarding this feature: some experience being named as genuine, others as disingenuous. Additionally, if the sender is known and identifiable, his could influence the participant’s trust in and perception of the credibility and value of the intervention and the information it provides, and strengthen the belief that the messages were sent by a person, even if sent from an automated service.^26^ However, some patient groups, for example with stigmatized health conditions, may prefer an unmarked sender to protect privacy. Guidance grounded in research and best practice increase users’ confidence to participate and their perceptions of expertise and trustworthiness is likely to help strengthened the therapeutic alliance.^29^ The tone of the intervention content delivered via mobile devices are of importance.^38^ There is consensus across studies that digital interactions should be polite and respectful. The tone used in the mHealth intervention has been found to influence acceptance of the program, the trust and the credibility of content, and participants’ engagement in the intervention.^33^ In addition, aspects such as text messages without ‘textese’ that instead are written out and do not use slang, are preferrable as these feels more professional and are more representative of how health professionals would write.^33,39^ As in face-to-face meetings, people’s preferences vary, but participants reported preferring a tone that is, for example, motivational, personalized, encouraging, polite, respectful, friendly, positive, supportive, and relatable.^33^

### Future research

Future studies need to continue to explore design considerations, both technological and human-like, that may enhance the digital encounters, taking personal preferences and contextual factors into account.^33^ Developers of mHealth interventions should pay attention to the fact that tailoring and personalizing content may help foster the digital therapeutic alliance^29^ and that a tone that is polite and respectful might influence acceptance, trust, and engagement in the intervention.^33,35,38^ In unsupported mHealth interventions, it may be even more important to develop interventions in which the experience of human presence is present. AI chatbots can simulate human face-to-face communication. Chatbots can build relationships with users by means of speech, text, or both, and can be deployed in mHealth interventions, e.g. in the form of mobile apps.^40^ Chatbots are designed based on data of individual characteristics and behavior trajectories.^41^ The design of just-in-time adaptive interventions^34^ in combination with personal preferences and contextual factors allow chatbots to customize the timing, amount, content, and frequency of the intervention by adapting each individual’s internal and external changes over time.^40,41^ These technological features (timing, amount, content, and frequency) are similar to the human-like qualities intended to enhance digital encounters identified in previous research on client’s perceptions of digital communication.^29,33,37^ As in the therapeutic alliance, chatbots are design to achieve behavior change goals, and deliver customized, personalized treatment^34^ and chatbots tend to be rated positively on empathy and alliance.^40^ Consequently, chatbots might have the potential to an alternate route to facilitate the digital encounters and may be deemed suitable to adolescents, who are familiar with smartphones.^8^ More research is required to understand the feelings associated with digital interactions and treatment outcomes, and the role of therapeutic alliance as well as engagement in mHealth interventions. Gaining at better understanding of the function of emotion within user experiences is critical to comprehending digital encounters in unsupported mHealth interventions, chatbots included, as emotions is closely tied to user satisfaction, and influence motivation for behavior change.

## Conclusions

Research is ongoing, but both empirical and theoretical gaps exist in the literature concerning digital interactions in mHealth interactions and their relationship to behavior change. This paper shared light to users’ perceptions of interacting within digital interventions and the complexity of the therapeutic alliance within mHealth. Several design considerations, both technological and humanoid, are highlighted aiming to enhance the digital encounters. The awareness gained from this paper may be valuable in the process to continue developing effective human-to-human conversations in interventions to improve health behaviors.
